# Simulation of FEL pulse length calculation with THz streaking method

**DOI:** 10.1107/S160057751600285X

**Published:** 2016-04-02

**Authors:** I. Gorgisyan, R. Ischebeck, E. Prat, S. Reiche, L. Rivkin, P. Juranić

**Affiliations:** a Paul Scherrer Institut, 5232 Villigen PSI, Switzerland; b École Polytechnique Fédérale de Lausanne, Route Cantonale, 1015 Lausanne, Switzerland

**Keywords:** simulation, free-electron laser, femtosecond metrology, terahertz streaking, hard X-rays, time-of-flight spectrometer

## Abstract

Having accurate and comprehensive photon diagnostics for the X-ray pulses delivered by free-electron laser (FEL) facilities is of utmost importance. Along with various parameters of the photon beam (such as photon energy, beam intensity, *etc*.), the pulse length measurements are particularly useful both for the machine operators to measure the beam parameters and monitor the stability of the machine performance, and for the users carrying out pump–probe experiments at such facilities to better understand their measurement results. One of the most promising pulse length measurement techniques used for photon diagnostics is the THz streak camera which is capable of simultaneously measuring the lengths of the photon pulses and their arrival times with respect to the pump laser. This work presents simulations of a THz streak camera performance. The simulation procedure utilizes FEL pulses with two different photon energies in hard and soft X-ray regions, respectively. It recreates the energy spectra of the photoelectrons produced by the photon pulses and streaks them by a single-cycle THz pulse. Following the pulse-retrieval procedure of the THz streak camera, the lengths were calculated from the streaked spectra. To validate the pulse length calculation procedure, the precision and the accuracy of the method were estimated for streaking configuration corresponding to previously performed experiments. The obtained results show that for the discussed setup the method is capable of measuring FEL pulses with about a femtosecond accuracy and precision.

## Abstract 2

Simulation of THz streaking of photoelectrons created by X-ray pulses from a free-electron laser and reconstruction of the free-electron laser pulse lengths.

## 1. Introduction   

Free-electron lasers are able to produce short pulses of radiation in both soft and hard X-ray regions with a typical radiation power of about 10 GW (with pulse lengths in range of 10 fs), which is orders of magnitude higher than the third-generation synchrotron light sources. Typical experiments carried out at FEL facilities around the world (Ackermann *et al.*, 2007 ▸; Emma *et al.*, 2010 ▸; Ishikawa *et al.*, 2012 ▸) are pump–probe experiments where the sample is pumped by the experiment laser and then probed by the FEL beam. The resolution of pump–probe experiments is mostly dependent on the pumping and probing pulse lengths and the stability of the time delay between these two pulses. In order to improve the resolution of such experiments, to set up the machine itself and monitor its stability, it is important to measure the temporal properties of the photon pulses (pulse duration and the arrival time) on a shot-to-shot basis. Providing accurate photon diagnostics at femtosecond timescales is, however, challenging.

In recent years various methods have been proposed and developed for accurate measurements of relative arrival times (Bionta *et al.*, 2011 ▸; Tavella *et al.*, 2011 ▸; Düsterer *et al.*, 2011 ▸; Harmand *et al.*, 2013 ▸; Hartmann *et al.*, 2014 ▸; Juranić *et al.*, 2014*a* ▸) and pulse lengths (Drescher *et al.*, 2001 ▸; Frühling *et al.*, 2009 ▸; Ding *et al.*, 2011 ▸; Düsterer *et al.*, 2011 ▸; Inubushi *et al.*, 2012 ▸; Grguraš *et al.*, 2012 ▸; Riedel *et al.*, 2013 ▸). Among these methods, the THz streak camera (Itatani *et al.*, 2002 ▸; Frühling *et al.*, 2009 ▸; Grguraš *et al.*, 2012 ▸; Helml *et al.*, 2014 ▸; Juranić *et al.*, 2014*b* ▸) is able to measure both the length and the arrival time of the photon pulses at wavelengths ranging from UV to hard X-ray.

A THz streak camera called the pulse arrival and length monitor (PALM) (Juranić *et al.*, 2014*b* ▸) has been developed at the Paul Scherrer Institute (PSI) to provide online single-shot diagnostics for the FEL pulses delivered by the future SwissFEL facility (Ganter, 2010 ▸; Oberta *et al.*, 2011 ▸). A prototype PALM setup was built and tested (Juranić *et al.*, 2014*a* ▸) both at PSI and at the SPring-8 Angstrom Compact free-electron LAser (SACLA): a hard X-ray FEL facility in Japan (Ishikawa *et al.*, 2012 ▸; Tono *et al.*, 2013 ▸).

To better understand the measurements from the PALM setup and THz streak cameras in general, to improve the data analysis procedure used in the measurements and to validate the theory used in THz streak cameras for photon pulse length retrieval, a Matlab simulation code was developed for streaking and pulse length calculation. The code operates as follows: it takes as an input FEL pulses generated externally by the code *Genesis* (Reiche, 1999 ▸), and simulates the energy streaking of the photoelectrons produced by these pulses. For generating the electron spectra and simulating the streaking effect, a Monte Carlo method called rejection sampling (acceptance–rejection) was used (Gilks & Wild, 1992 ▸; Robert & Casella, 2013 ▸). The code then calculates the photon pulse lengths following the procedure used in a THz streak camera.

Once the pulse lengths are delivered by the simulation, the accuracy and the precision of the measurement technique is estimated for various pulse lengths. The accuracy in this work is defined as the absolute difference between the initial length of the photon pulse and the pulse length obtained by the simulation, whereas the precision is used as the standard deviation of the calculated values obtained by a set of simulations for the same pulse length.

## 2. Concept   

The theory of the THz streak camera and the photon pulse length calculation method is explained well in the works by Itatani *et al.* (2002 ▸), Uiberacker *et al.* (2005 ▸) and Quéré *et al.* (2005 ▸). This section provides a short summary of the concept mostly following the derivations given by Frühling *et al.* (2009 ▸) and Juranić *et al.* (2014*b* ▸). The main idea of the concept is to encode the temporal properties of the photon pulse in the energy spectrum of the photoelectrons created by the pulse. This is achieved by overlapping the photoionization region with an external time-varying electromagnetic field. In a THz streak camera, the photon pulse propagates through a gas ionizing an ensemble of electrons whose energies are streaked depending on their emission times with respect to the THz pulse. The kinetic energy of an electron produced by a photon without an external field is defined only by the incident photon energy and the properties of the atom being ionized. When the electron is created in the presence of an external THz field, it starts interacting with the field of the THz pulse and an energy exchange between the electron and the field takes place. The streaking field is taken as a linearly polarized THz wave with an electric field strength given as 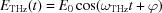, where 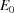 is the field amplitude, *t* is the time, 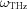 is the THz frequency and φ is a phase constant. For the electron travelling in the plane of polarization of the THz, the final kinetic energy after the interaction is given by
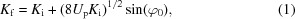
where 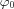 is the phase of the electric field at the instant of the ionization and 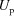 is the ponderomotive potential. In the derivation of equation (1) it is assumed to be small, 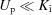, and it is given by the following expression:
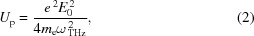
where *e* and 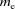 are the electron charge and mass, respectively. Equation (1) shows that the final kinetic energy of a photoelectron propagating in an external THz field depends on the moment of the electron emission with respect to the THz pulse. As the electrons ionized by different parts of the photon pulse are created at different times, they experience different field strengths of the THz field and, therefore, end up with different kinetic energies. The final energy spectrum of the photoelectrons created in the external time-varying field is a convolution of their energy spectrum without the streaking field and the temporal profile of the ionizing photon pulse. In the case of convolution, the root mean square (r.m.s.) widths add up in quadrature and the square of the r.m.s. width of the streaked electron spectrum can be written as
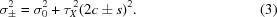
Here, 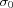 is the r.m.s. width of the electron spectrum without the external streaking field or any energy chirp, 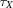 is the r.m.s. length of the photon pulse, and the terms *s* and *c* are the streaking strength and the linear photon energy chirp along the FEL pulse, respectively. The two opposite signs in equation (3) correspond to two opposite directions of the electrons propagating along the streaking field and opposite to it. The change of the spectral width of the electrons with the streaking strength can be written as 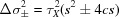. Therefore, by simultaneously measuring this difference of the streaked and non-streaked spectra for two opposite streaks, one can exclude the term containing the chirp and the r.m.s. length of the photon pulse can be extracted:
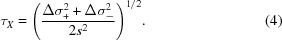
In this equation, 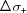 and 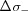 are the r.m.s. spectral width differences for the photoelectrons experiencing two opposite streaks. Equation (4) shows that it is possible to reconstruct the photon pulse length by simultaneously measuring the energy spectra of the non-streaked electrons and the electrons streaked in two opposite directions. This procedure is used in the simulations for the pulse length retrieval.

## 3. Simulation   

The main goal of the simulation is to reproduce the THz streaking effect and obtain energy spectra of the photoelectrons for the experimental setup used in pulse length measurements. The delivered spectra allow the validation of the analysis procedure used in a THz streak camera and estimate the precision and the accuracy of the method. The simulation utilizes FEL pulses generated by *Genesis* for SwissFEL parameters.

### 3.1. Simulation model   

The simulation models the photoionization process and the energy streaking of the electrons in a simple way, concentrating only on the phenomena that are relevant for the photon pulse length measurement application. For example, effects such as double-photon ionization, Gouy phase shift, *etc*. are not discussed in the model. As the average duration of the photoionization process is comparable with the coherence time of the FEL [of the order of 100 attoseconds for the pulses discussed here], the energy spectrum of the photoelectrons does not simply mirror the spectrum of the photon pulse; it also has a contribution from the ionization emission spectrum. The latter refers to a Gaussian energy spectrum which is the Fourier transformation of the Gaussian profile of the mean ionization time of the photoelectrons (Schultze *et al.*, 2010 ▸). Depending on this time, any of the two components can be dominant in the final spectrum of the electrons. When the mean ionization time is faster than the coherence time of the photon pulse, the spectrum of the photoelectrons is broader than the original spectrum of the photons and it is dominated by the ionization emission spectrum. Most of the studies about the mean ionization time carried out so far refer to the outer shell electrons using photon energies of a few hundred electronvolts and report values from a few tens up to hundreds of attoseconds (as) (Dahlström *et al.*, 2012 ▸, 2015 ▸; Kheifets, 2013 ▸; Guénot *et al.*, 2014 ▸). A recent study of mean emission time of the inner shell electrons with photon energies of up to 10 keV has obtained delays of about 10 as (Kheifets *et al.*, 2015 ▸). For such values of time the spectrum of the photoelectrons should be dominated by the photoionization emission spectrum. In the case of a 10 as emission time the corresponding energy spectrum is very broad and requires many sampling points in the simulation, which makes the procedure slow. For this reason, the simulation model takes the ionization mean time from the inner shells to be about 50 as which still keeps the emission the dominant component of the observed spectra and does not make the simulation process too bulky as it would be for the value of 10 as. Choosing a mean emission time of 50 as instead of 10 as changes only the width of the energy spectrum of the photoelectrons. This change does not affect the final result for the photon pulse length as during the simulation procedure the energy spectrum is first convoluted to the temporal profile of the photon pulse during the streaking and then deconvoluted back during the pulse length calculation process.

**Figure 1:**
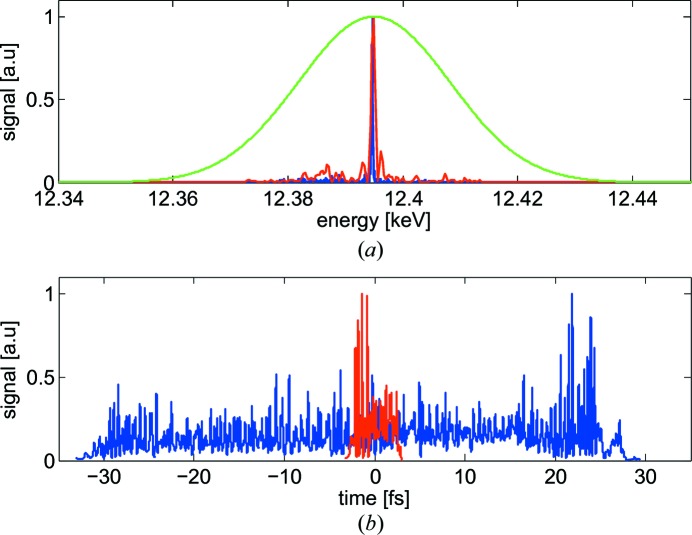
Energy spectra (*a*) and temporal profiles (*b*) of two photon pulses for SwissFEL long-pulse (blue) and short-pulse (red) operation modes and the Gaussian spectrum corresponding to a 50 as mean ionization time (green).

Fig. 1 ▸(*a*) shows the spectra of two photon pulses with different lengths and the spectrum corresponding to a 50 as ionization time. This spectrum is dominant for all the pulses used in the simulation and, therefore, the energy spectra of the non-streaked photoelectrons are always considered to have the same Gaussian profile.

This simulation utilizes a THz pulse with a frequency of 0.5 THz and a peak electric field strength of 6 MV m^−1^ corresponding to the THz pulse generated during the experiments with the PALM setup (Juranić *et al.*, 2014*a* ▸). For this experimental setup the uncertainty in the pulse length measurement is mainly caused by the limited number of the detected photoelectrons and the energy resolution of the electron time-of-flight spectrometers (eTOFs) used in the measurements (about 1.2 eV). In the above-mentioned experiment the arrival time jitter of the photon pulses with respect to the THz pulse was about 100 fs r.m.s., whereas the linear part of the THz pulse was more than 600 fs long. This means that the streaking strength [term *s* in equation (4)] could be considered equivalent for all the photon pulses, even for those arriving away from the zero-crossing of the THz. For this reason, the jitter of the arrival time of the photon pulses relative to the THz pulse is not considered in this simulation and all the pulses are assumed to arrive at the zero-crossing of the THz pulse. Based on the same measurements, the electric field jitter of the THz pulse is also neglected and it is assumed constant in the simulations.

The central energies of the photoelectrons without the streaking field are taken as half of the photon energies. This corresponds to 6.2 keV and 0.62 keV of initial central energies of the electrons for hard and soft X-rays, respectively. The chosen values for binding energies used in the simulation are realistic and are comparable with the relevant binding energies of the noble gases used in streak cameras.

This simulation model assumes that the number of the photoelectrons registered by the eTOFs is in range from a few thousands up to 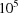, depending on the length of the photon pulse. These numbers were chosen based on the measurements performed by the PALM detector (Juranić *et al.*, 2014*a* ▸,*b* ▸). A study carried out by Wellhöfer *et al.* (2008 ▸) discusses the space-charge effect in the photoelectron spectra created by FEL pulses. For about 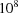 photoelectrons with energies of 15–68 eV, the contribution of the space-charge effect in the electron energy spectra is only about 1 eV. For the case discussed in this work the maximum number of electrons registered by the eTOFs is 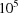, corresponding to about 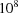 electrons created by the photon pulse at the interaction region. This quantity of the electrons is the same as reported by Wellhöfer *et al.* (2008 ▸), whereas the kinetic energies are much higher (0.62 keV or 6.2 keV), making the space-charge effect significantly smaller than 1 eV. As the resolution of the eTOF detectors is about 1.2 eV, the space-charge effect can be neglected in the simulation.

### 3.2. Simulation of FEL pulses   

The FEL process was simulated with the code *Genesis* (Reiche, 1999 ▸). The electron beam properties and the lattice parameters have been chosen based on the SwissFEL specifications (Ganter, 2010 ▸). Overall, 178 photon pulses were produced with r.m.s. durations varying between about 1 fs and 15 fs for the radiation wavelength of 0.1 nm and between 20 fs and 40 fs for 1 nm wavelengths. Some of the simulations were carried out with the standard SASE (Kondratenko & Saldin, 1980 ▸; Bonifacio *et al.*, 1984 ▸) configuration, whereas some others include the option of self-seeding (Feldhaus *et al.*, 1997 ▸; Saldin *et al.*, 2001 ▸; Geloni *et al.*, 2010 ▸; Amann *et al.*, 2012 ▸) to reduce the bandwidth of the FEL pulse. Fig. 1 ▸ shows the time profiles and spectra of two of the simulated FEL pulses for a 0.1 nm wavelength. The blue and the red curves in the figure show the long-pulse and the short-pulse configurations, respectively, of the SwissFEL.

### 3.3. Simulation procedure   

The simulation procedure commences once a photon pulse is generated with a defined temporal profile and a defined energy spectrum. Photoelectron spectra are generated and the streaking is simulated in two opposite directions corresponding to the electrons propagating along the streaking electric field and opposite to it. After obtaining the streaked spectra of the photoelectrons and using their non-streaked spectrum, the r.m.s. photon pulse length is calculated following the standard analysis procedure based on equation (4).

**Figure 2:**
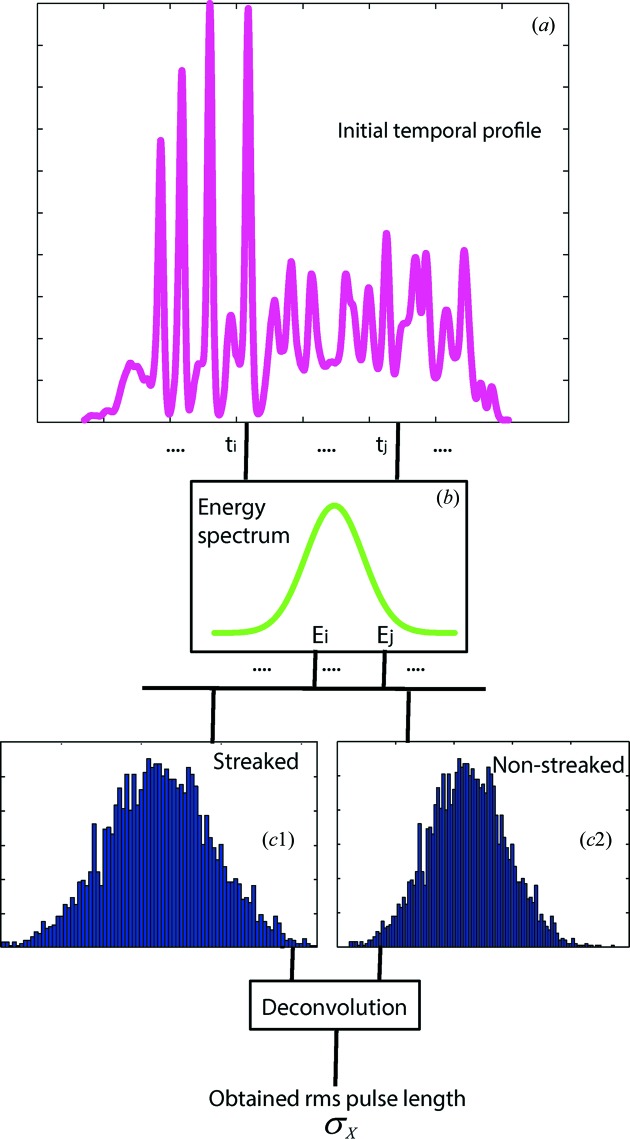
Diagram showing the simulation procedure. Random points are chosen from the temporal profile (such as 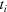 and 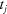) of the photon pulse (*a*). When an electron is created at a particular point of the profile an energy value (such as 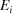 or 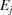) from the spectral distribution is assigned to it (*b*). Combining the energy values from all the created photoelectrons provides the streaked and non-streaked energy spectra (*c*1 and *c*2, respectively). Deconvolution of these two gives the r.m.s. duration of the photon pulse.

At the beginning of the simulation, a number of photoelectrons are generated depending on the length of the ionizing photon pulse. Here, the number of photons per unit length of the pulse is considered the same for all the pulses and, therefore, the number of created photoelectrons is taken as proportional to the pulse lengths. Each photoelectron is simulated by the rejection sampling method. The procedure of the simulation is illustrated in the diagram in Fig. 2 ▸. First, a random point 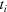 is taken along the temporal profile of the photon pulse which corresponds to the position of the photon field that would ionize the electron. Another random number *a* is generated between 0 and 1 that defines whether the photoionization at the selected point 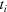 should be considered or not. The number is compared with the value of the normalized profile 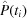 = 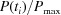 at the taken point. If the amplitude 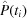 is bigger than the generated number *a*, the chosen point is accepted and a photoelectron is produced from the position 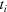 of the photon pulse. This procedure is repeated until the required amount of photoelectrons is created. The initial non-streaked energy of each electron is randomly generated from the Gaussian distribution of the emission spectrum: 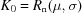, where *R*_n_ represents a function generating random numbers with a normal distribution. The mean value μ in the function is the central energy equal to the mean energy of the photon pulse minus the binding energy: 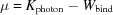. The r.m.s. width σ corresponds to the 50 as ionization time.

When the final spectrum of the electrons is registered by the eTOF spectrometer, its acceptance function should be convoluted with the spectrum. It is added in the expression for the energy of a non-streaked electron detected by the spectrometer:
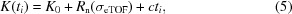
where 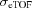 is the resolution of the spectrometer, whereas the term 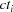 is the contribution from a linear chirp along the photon pulse. As the photon pulses delivered by the FEL facilities may have a linear energy chirp along their temporal profile, it is included in the simulation to check the effect of the chirp in the pulse length calculations. The chirp *c* in the equation is defined as the change of the central energy of photons along the pulse per unit time. It is used in calculations in units of meV fs^−1^. The effect of the nonlinear chirp is not discussed in this simulation as this effect is typically negligible at FEL facilities.

Analogously, the final kinetic energy of an electron that is streaked in the THz field can be written as
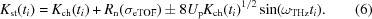
The term 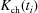 here is the kinetic energy of the electron before streaking, including also the effect from the linear chirp. The last term on the right-hand side is the energy streaking in accordance with equation (1).

**Figure 3:**
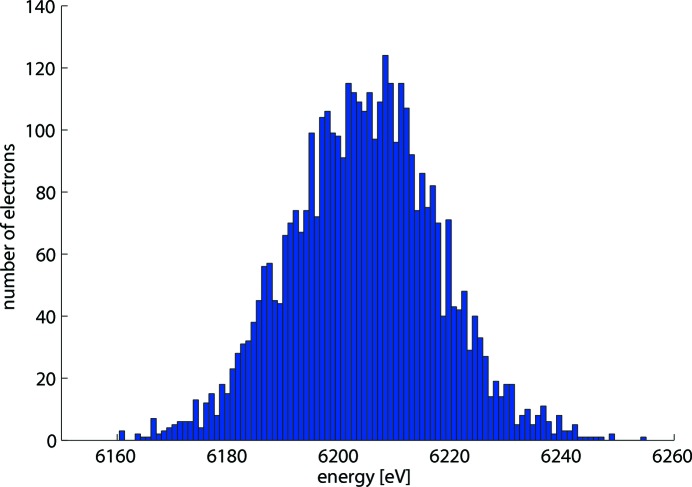
Energy distribution of the photoelectrons created by a femtosecond-long photon pulse, registered by an eTOF spectrometer.

Based on equations (5) and (6), energy values are generated and assigned to the number of photoelectrons produced by each photon pulse. As the non-streaked spectrum and the two streaked spectra are measured independently by different detectors, a new set of random numbers is generated for each of these spectra. This ensures that the effect of statistical fluctuations of the spectra is included in the pulse length calculation procedure. Fig. 3 ▸ shows the distribution of the electron energies registered by an eTOF spectrometer. These energy values are generated by the procedure described above and correspond to photoelectrons created by a photon pulse of 1.5 fs at a photon energy of 12.4 keV, without external streaking. One can see from the figure that the spectrum is not a smooth Gaussian due to the small number of electrons created by a short pulse. After recreating the electron spectra from the simulations, the r.m.s. pulse length can be calculated from equation (4) using the spectral width differences 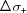 and 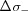.

As the quantities 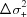 and 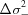 are defined as the quadratic difference of the streaked and the non-streaked spectra, they may have also negative values when the statistical fluctuations of the spectra are larger than the streaking itself. This may result in a negative sign under the square root in equation (4) making the pulse length value imaginary. Such a result is an artifact of the evaluation process and does not have any physical meaning. For this reason the simulation code has to check and reject these imaginary values that appear, especially in the case of short photon pulses where the difference between the streaked and non-streaked spectral widths is small. When the non-physical values are filtered out, the distribution of the remaining values is no longer symmetric around the mean value and has a cut-off at the zero length. This skews the final value of the average pulse length shifting it towards higher numbers. This means that the acceptance rate of a calculation set can also affect the accuracy of the results. This effect is observed in the simulations for short pulses and is discussed in the next section. In order to have a better estimation of a pulse length in average after many shots, one can keep the square value of the pulse lengths including also the negative ones. In this case the distribution of the obtained values will be symmetric around the mean and the square root of this mean value will describe the pulse length more accurately. The simulation procedure in this paper concentrates on single-shot measurements filtering out the imaginary values and the calculated mean pulse lengths give more the upper limit for the short pulses rather than measuring the actual values.

The r.m.s. spectral widths used for the pulse length calculation were obtained in two different ways. The first way was to perform a Gaussian fit to the spectrum and take the standard deviation value of the fit as the r.m.s. width of the spectrum. This fitting procedure is the one most commonly used in the data analysis and the theory of the THz streak camera is developed for Gaussian pulses (Itatani *et al.*, 2002 ▸; Frühling *et al.*, 2009 ▸). The second method used for the pulse length calculation was to evaluate the standard deviation of the spectrum directly from the photoelectron energy distribution (shown in Fig. 3 ▸). This method does not depend on the shape of the electron spectra and can provide accurate results even for non-Gaussian spectra. However, it is less often used in the data analysis as the spectra registered during the measurements also have a background signal from other electrons (from other shells or Auger electrons) and defining the precise range for the spectrum of a particular line for each photon pulse is challenging.

After calculating the pulse lengths following the simulation procedure described above, the obtained results are compared with the initial pulse lengths.

## 4. Results   

The r.m.s. duration of the used 178 different FEL pulses was in the range from about 1 fs up to 40 fs. The pulses had photon energies of 1.24 keV or 12.4 keV. The lengths of the pulses were calculated following the procedure described in §3.3. The spectral widths were obtained by either performing Gaussian fits to the spectra or taking their r.m.s. widths directly. These two methods revealed different results in terms of precision and accuracy.

The results obtained by fitting Gaussian profiles to the energy spectra are presented in Table 1 ▸ for some photon pulses randomly chosen per pulse length. The initial r.m.s. lengths of the pulses and the mean lengths delivered by the simulation are shown as well as the standard deviation of the pulse length from 100 measurements per input pulse and the accuracy of the mean value. The difference between the hard and soft X-ray pulses is that the energy of the created electrons before streaking is different and, therefore, they are streaked by different amounts according to equation (6): the higher the initial energy of the electrons the more is the absolute streak of the spectra. This means that if both types of electrons are detected by the eTOFs with the same resolution, then the spectra which are more streaked should give better accuracy and precision. Such a result can be observed in Table 1 ▸, which shows better agreements for the more energetic 6.2 keV electrons produced by the hard X-ray pulses.

**Figure 4:**
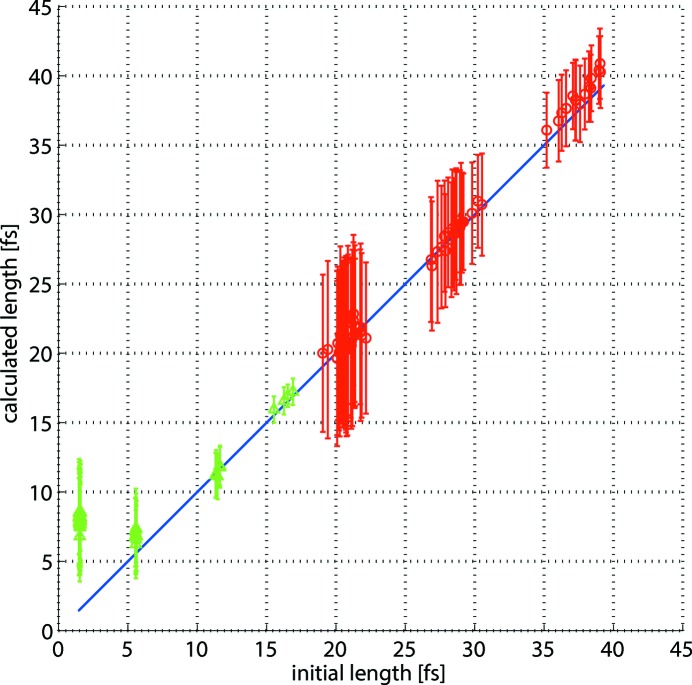
Simulation results obtained by Gaussian fitting. The blue diagonal line represents the initial r.m.s. pulse lengths, the green triangles and the red circles are the calculated average lengths for photon energies of 12.4 keV and 1.24 keV, respectively. The vertical bars correspond to the standard deviations from 100 shots.

For short photon pulses the amount of imaginary values for the pulse length that occur due to statistical fluctuations of the spectra is about 50%. As these non-physical results are rejected by the simulation, the average pulse length is shifted towards higher values which can be seen in Table 1 ▸. For the pulses of about 5 fs r.m.s. the acceptance rate is about 75% and it reaches 100% for the longer pulses. The comparison of the results delivered by the simulations and the initial pulse lengths is illustrated in Fig. 4 ▸. Here the blue diagonal reference line shows the initial pulse lengths, whereas the green triangles and the red circles correspond to the calculated pulse lengths for hard and soft X-ray photons, respectively. The vertical bars in the figure represent the precision of the calculation which is the standard deviation of a set of calculations for each photon pulse. It changes from about 6 fs down to sub-fs. The precision is better for hard X-ray photon pulses as they correspond to stronger streaking. It also improves with longer pulses as the number of created photoelectrons is higher in this case and, therefore, the statistical fluctuations are smaller, making the calculations more reproducible for each single shot.

The accuracy of the 1.5 fs photon pulses is about 6 fs providing the upper limit of the pulse length values. This is caused by the high rejection rates of non-physical pulse lengths. For the pulses of 5 fs and longer the accuracy is better than 2 fs down to sub-fs. One can also see both from the figure and the table that the accuracy gets slightly worse for the longest pulses used in the simulations. The reason for such a result is that the spectra of the streaked photoelectrons from the long photon pulses are no longer Gaussian and the errors induced by the fitting process become significant. For such cases the second method of pulse length retrieval that uses the r.m.s. widths of the spectra provides better results.

The second way to evaluate the widths of the electron spectra is to calculate the r.m.s. widths directly from the simulated data. The spectral widths obtained by this method describe the spectrum better and are not dependent on the shape of the peaks, so the calculated pulse lengths are more accurate. The mean values obtained this way are shown in Table 2 ▸ with their standard deviations and accuracies for the same photon pulses as given in Table 1 ▸. As one can see from the table, the calculated mean lengths for the short pulses are shifted towards higher numbers. As in the previous case, such a result is caused by the high rejection rate of non-physical pulse length values (about 50%). About 85% of the pulses with r.m.s. length of 5 fs are accepted in the simulation and for longer pulses the acceptance is 100%. The accuracy of the calculated mean values is about 5 fs for the 1.5 fs-long pulses and is better than a femtosecond for all the other pulses.

**Figure 5:**
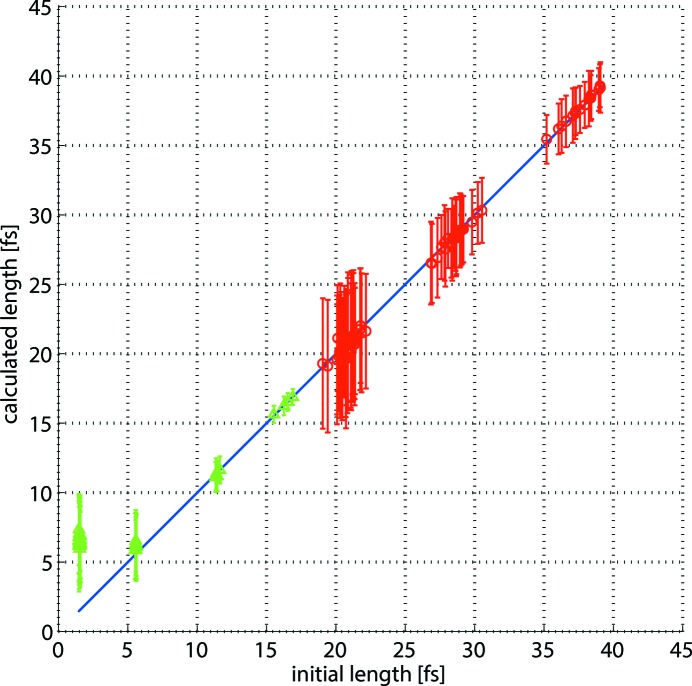
Simulation results obtained using the r.m.s. widths. The blue diagonal line represents the initial r.m.s. pulse lengths, the green triangles and the red circles are the calculated average lengths for photon energies of 12.4 keV and 1.24 keV, respectively. The vertical bars correspond to the standard deviations from 100 shots.

The results delivered by this method for all 178 photon pulses are displayed in Fig. 5 ▸. From the vertical bars in the figure one can see that the precision changes from about 5 fs down to sub-fs. It improves when moving towards longer pulses and higher photon energies which is consistent with the results delivered by the previous method.

## 5. Discussion   

**Figure 6:**
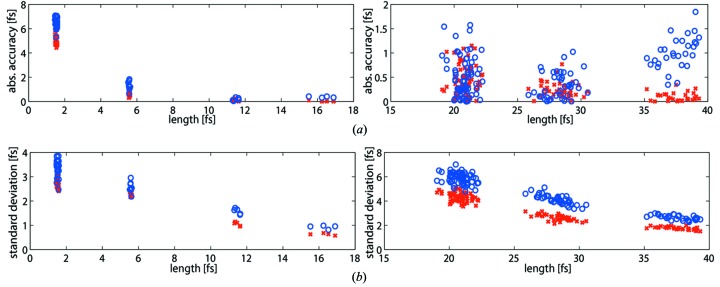
(*a*) Absolute accuracies of the mean pulse lengths obtained by the simulation and (*b*) the standard deviations from 100 simulations for each pulse length. The plots on the left show the results for 12.4 keV photon energies and the ones on the right for 1.24 keV. The blue circles correspond to the results obtained by Gaussian fitting whereas the red crosses show the results from the method using the r.m.s. widths.

The results obtained from the simulations help to characterize the THz streak camera measurement method and indicate possible ways to achieve the highest measurement accuracy for different pulse lengths by changing the streaking parameters. Fig. 6 ▸ shows the accuracy of the obtained pulse lengths (Fig. 6*a*) and the precision of the calculations (Fig. 6*b*) provided by two different ways of obtaining the spectral widths. The plots on the left and right sides correspond to the results from the pulses with photon energies of 12.4 keV and 1.24 keV, respectively. The accuracy for the pulses longer than 5 fs is better than 2 fs for the both calculation methods. A significant difference between the Gaussian fitting and the r.m.s. methods can be observed for the shortest and the longest pulses. In the case of short pulses, the number of the created electrons is not sufficient to recreate a smooth Gaussian profile of the energy spectrum (Fig. 3 ▸) and the Gaussian fit does not represent the spectrum well, whereas for the longer pulses the streaked spectra have a more flat-top shape than Gaussian. In such cases, directly calculating the r.m.s. widths of the spectra is preferable. The results in Fig. 6 ▸(*a*) show that for the experimental setup described by Juranić *et al.* (2014*a* ▸) the THz streak camera is able to measure photon pulses from 5 fs to 40 fs with an accuracy of about 1 fs, whereas for the 1.5 fs-long pulses it can provide an upper limit with about 6 fs accuracy.

From Fig. 6 ▸(*b*) one can observe an improvement of precision with longer pulses which produce more photoelectrons reducing the statistical fluctuations of the spectra from shot to shot. The figure also shows that the spread of the calculated values is smaller for hard X-ray photons corresponding to more streaking of photoelectrons compared with the soft X-ray case, even though the latter corresponds to longer pulses. This shows that the lack of photoelectrons can be compensated by stronger streaking fields, reducing the contribution of the statistical fluctuations in overall uncertainties of the calculations. The precision of calculations using Gaussian fitting is slightly worse than for the method with the r.m.s. widths. The reason for this is that the fitting process introduces an additional error in the calculations from shot to shot.

The simulation results show that Gaussian fitting can be applied in most of the cases when the electron spectra have Gaussian shape. This method is easier to implement during the data analysis of the measurements and does not induce a significant error in the case of the mentioned setup. On the other hand, when the electron spectra are not Gaussian the method with the r.m.s. width should be utilized for more accurate results.

Fig. 6 ▸ indicates better accuracy and precision for more energetic electrons (created by the 12.4 keV photons) as they correspond to more absolute streaking. Such a result was obtained assuming that the eTOFs measure both types of the electrons with the same resolution. However, when the energy of the photoelectrons is too high, they will not be detected by the eTOFs with sufficient resolution which will affect the pulse length measurement accuracy. For this reason, to achieve better accuracy and precision it is preferable to increase the streaking field of the THz pulse instead of using more energetic electrons.

The simulation procedure concentrates only on the effects caused by the statistical fluctuations of the photoelectron spectra and by the limited resolution of the eTOF spectrometers used in the experiments. These effects are dominant for the present setup as described by Juranić *et al.* (2014*b* ▸,*a* ▸). It is possible to reduce the uncertainties and errors caused by these effects by increasing the streaking strength and the number of created photoelectrons. In this case, however, other sources of uncertainties may become dominant: for strong streaking field, for instance, small fluctuations of the field strength may cause large errors in the measurements, which implies stricter stability requirements for the streaking THz pulse. In the case of using higher gas densities to produce more photoelectrons, the space-charge effect may become more than an electronvolt causing a detectable broadening of the spectrum which will affect the final results. Bearing this in mind, it is possible to alternate some parameters of the experimental setup to achieve a measurement accuracy of a femtosecond and better at any given pulse length.

As this simulation does not restrict itself to a specific temporal profile of FEL pulses, the obtained results are valid not only for SwissFEL but also for any other FEL facility delivering SASE or self-seeded photon pulses with pulse lengths from 1 fs to 40 fs.

## 6. Conclusion   

A simulation procedure was developed to model the THz streaking process and calculate the photon pulse lengths following the pulse retrieval method of the THz streak camera. The simulation used 178 FEL pulses with r.m.s. lengths from about 1 fs to 40 fs, most of them corresponding to SwissFEL standard operation modes. The pulse lengths obtained through the simulation procedure were compared with the initial lengths of the pulses calculated directly from the temporal profiles. Two different ways of calculating the spectral widths were discussed: performing Gaussian fits to the energy spectra of the electrons or calculating the r.m.s. widths of the spectra directly. For most of the pulses used in the simulation both methods provided similar results without a significant difference. However, in some cases the method using the r.m.s. widths of the spectra was shown to be more accurate, even though being more difficult to implement in the data analysis process. The accuracy of the calculation was about 1 fs for the 5 fs pulses and longer. For the pulses of about 1 fs the obtained values gave only an upper limit due to the high rejection rate in the simulation procedure. The obtained results provide a good estimate of the accuracies to be expected from the measurements of different FEL pulse lengths using a THz streak camera.

## Figures and Tables

**Table 1:** Photon pulse lengths calculated by performing Gaussian fits Mean values of the obtained lengths (calculated mean), with standard deviations and accuracies compared with initial lengths.

Photon energy	Initial length (fs)	Calculated mean (fs)	Standard deviation (fs)	Accuracy (fs)
12.4 keV	1.5	8.2	3.7	6.7
	1.6	8.5	3.5	6.9
	5.6	6.6	2.5	1.0
	11.6	11.9	1.4	0.3
	15.5	15.9	0.9	0.4
1.24 keV	19.1	20	5.7	0.9
	22.2	21.8	5.5	0.4
	25.9	26.0	4.6	0.1
	30.5	30.7	3.7	0.2
	35.2	36.1	2.7	0.9
	39.3	40.6	2.5	1.3

**Table 2:** Photon pulse lengths calculated using the r.m.s. widths of the spectra Mean values of the obtained lengths (calculated mean), with standard deviations and accuracies compared with initial lengths.

Photon energy	Initial length (fs)	Calculated mean (fs)	Standard deviation (fs)	Accuracy (fs)
12.4 keV	1.5	6.2	2.8	4.7
	1.6	6.3	2.8	4.7
	5.6	5.9	2.3	0.3
	11.6	11.7	0.9	0.1
	15.5	15.6	0.6	0.1
1.24 keV	19.1	19.3	4.7	0.2
	22.2	21.7	4	0.5
	25.9	26.1	3.1	0.2
	30.5	30.3	2.3	0.2
	35.2	35.4	1.8	0.2
	39.3	39.2	1.5	0.1
